# HLA class II antibodies induce necrotic cell death in human endothelial cells via a lysosomal membrane permeabilization-mediated pathway

**DOI:** 10.1038/s41419-019-1319-5

**Published:** 2019-03-08

**Authors:** Abid Aljabri, Vijith Vijayan, Metodi Stankov, Christoph Nikolin, Constanca Figueiredo, Rainer Blasczyk, Jan Ulrich Becker, Andreas Linkermann, Stephan Immenschuh

**Affiliations:** 1 Institute for Transfusion Medicine, Hannover Medical School, Hannover, Germany; 2 King Saud Medical City, Riyadh, Saudi Arabia; 3 Department for Clinical Immunology and Rheumatology, Hannover Medical School, Hannover, Germany; 4 Institute of Pathology, University Cologne, Cologne, Germany; 5 Department of Internal Medicine III, Division of Nephrology, University Carl Gustav Carus, Dresden, Germany

## Abstract

Antibody-mediated rejection (AMR) is the major cause of allograft loss after solid organ transplantation. Circulating donor-specific antibodies against human leukocyte antigen (HLA), in particular HLA class II antibodies are critical for the pathogenesis of AMR via interactions with endothelial cells (ECs). To investigate the effects of HLA class II antibody ligation to the graft endothelium, a model of HLA-DR antibody-dependent stimulation was utilized in primary human ECs. Antibody ligation of HLA class II molecules in interferon-γ-treated ECs caused necrotic cell death without complement via a pathway that was independent of apoptosis and necroptosis. HLA-DR-mediated cell death was blocked by specific neutralization of antibody ligation with recombinant HLA class II protein and by lentiviral knockdown of HLA-DR in ECs. Importantly, HLA class II-mediated cytotoxicity was also induced by relevant native allele-specific antibodies from human allosera. Necrosis of ECs in response to HLA-DR ligation was mediated via hyperactivation of lysosomes, lysosomal membrane permeabilization (LMP), and release of cathepsins. Notably, LMP was caused by reorganization of the actin cytoskeleton. This was indicated by the finding that LMP and actin stress fiber formation by HLA-DR antibodies were both downregulated by the actin polymerization inhibitor cytochalasin D and inhibition of Rho GTPases, respectively. Finally, HLA-DR-dependent actin stress fiber formation and LMP led to mitochondrial stress, which was revealed by decreased mitochondrial membrane potential and generation of reactive oxygen species in ECs. Taken together, ligation of HLA class II antibodies to ECs induces necrotic cell death independent of apoptosis and necroptosis via a LMP-mediated pathway. These findings may enable novel therapeutic approaches for the treatment of AMR in solid organ transplantation.

## Introduction

Transplant rejection is the key limiting factor for the success of solid organ transplantation, which is determined by various immunologic and non-immunologic factors^1,2^. Antibody-mediated rejection (AMR) has been recognized as the major cause of allograft loss in kidney and heart transplantation^3–6^ and is primarily mediated by donor-specific antibodies (DSAs) against molecules of the major histocompatibility complex (MHC), synonymous with human leukocyte antigen (HLA) in humans^7,8^. Studies in animal models have revealed that MHC antibodies can cause transplant rejection in the absence of T cells^9,10^. Moreover, ligation of HLA antibodies to the endothelium of transplanted organs plays a critical role for the pathogenesis of AMR^11–13^. Principally, antibody-mediated injury in allografts is mediated via complement-dependent and -independent pathways^11,14–16^. Complement-dependent antibody-mediated damage appears to be mainly due to cytotoxicity via activation of the classical complement cascade by the Fc region of DSAs^14^. In contrast, complement-independent effects of DSAs are mediated via ligation with endothelial HLA molecules to induce intracellular signal transduction cascades^8,11^. Thus, it has been well established that ligation of HLA class I (HLA I) antibodies causes activation^17^ and leukocyte adhesion to ECs independent of complement^18,19^ (for reviews see refs. ^8,11^). In contrast to HLA I antibodies, much less is known on complement-independent effects of HLA II antibodies. For example, interleukin (IL)-6 secretion and cell proliferation have recently been shown to be upregulated by HLA II antibodies in ECs^20,21^. Notably, others have demonstrated that HLA II antibodies, such as the monoclonal antibody (mAb) L243 can cause cell death in the absence of complement in various types of non-adherent blood cells, such as leukemia cells^22,23^ and B cells^24^. Therefore, we hypothesized that HLA II antibodies may cause complement-independent cell death in human ECs.

Cell death, in particular regulated necrotic cell death, has emerged as a paradigm for the pathogenesis of numerous disorders, including inflammatory diseases^25–27^. In contrast to apoptosis, in which the plasma membrane remains intact, necrotic cell death is characterized by loss of plasma membrane integrity and subsequent release of pro-inflammatory damage-associated molecular patterns (DAMPs)^28^. The best characterized forms of regulated necrosis are necroptosis^29^ and ferroptosis^30^. Other forms of non-apoptotic cell death include pyroptosis, parthanatos, or cyclophilin D-mediated necrosis^25,26^. It is assumed that differences in the immunogenicity of cell death pathways may explain their evolutionary conservation^31^.

In the current report, we demonstrate that antibody ligation to HLA II molecules causes necrotic cell death in primary human ECs independent of complement. HLA-DR-dependent induction of EC death is primarily mediated via a pathway that involves reorganization of the actin cytoskeleton, lysosomal membrane permeabilization (LMP), and mitochondrial stress with generation of reactive oxygen species (ROS).

## Results

### Induction of necrotic cell death by HLA-DR antibody binding in cell cultures of human ECs

**Fig. 1:**
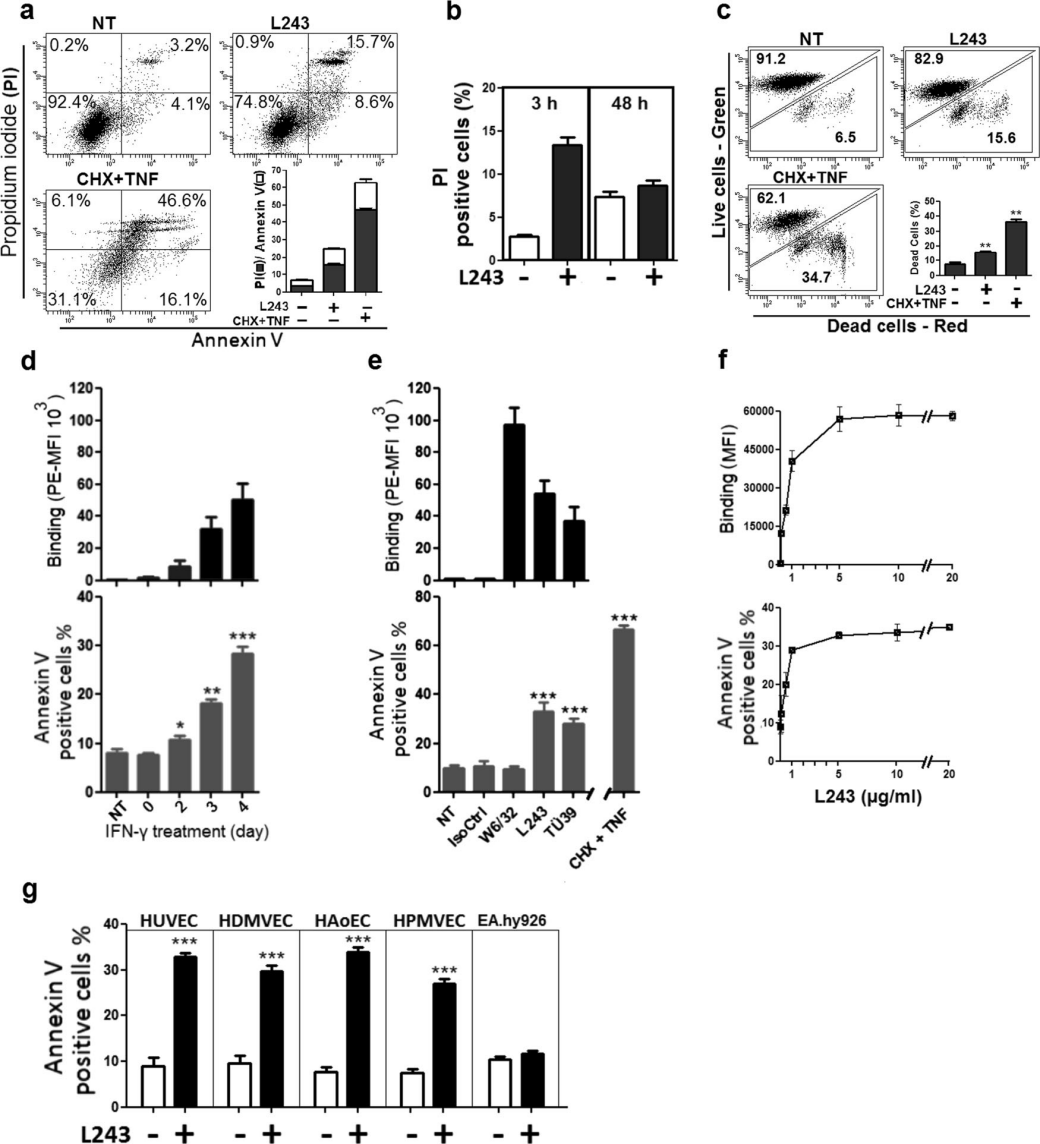
Antibodies against HLA-DR induce cell death in primary human ECs. **a** HUVECs were pretreated with 600 IU IFN-γ for 4 days, after which incubation was continued with HLA-DR mAb L243 (5 µg/ml) for 3 h. Cytotoxicity was assessed by flow cytometry with PI (■) and annexin V (□) (*N* = 3). **b** Detection of L243 cytotoxicity after 3 and 48 h in HUVECs pretreated with IFN-γ for 4 days (*N* = 4). **c** Cytotoxicity was assessed with LIVE/DEAD cell viability assay after 3 h of treatment with L243 (*N* = 3). **d** HUVECs were pretreated with IFN-γ for up to 4 days, after which incubation was continued with HLA-DR mAb L243 (5 µg/ml) for 3 h (*N* = 3). **e** After pretreatment with IFN-γ for 4 days, HUVECs were treated for 3 h with isotype control, the pan HLA I mAb W6/32 and the HLA-DR mAbs L243 or TÜ39 (5 µg/ml) as well as with CHX (10 µg/ml) plus TNF-α (50 ng/ml) (CHX + TNF) (*N* = 6). Cells were assessed for antibody binding levels and annexin V positivity by flow cytometry. Data are presented as mean ± SEM. ***P < 0.001. **f** After pretreatment with IFN-γ for 4 days, HUVECs were treated with increasing concentrations of L243 for 3 h. Annexin V positivity (lower panel) and antibody binding (upper panel) was determined by flow cytometry. Data are presented as mean ± SEM (*N* = 3). **g** After pretreatment with IFN-γ for 4 days, HUVECs, HDMVEC, HAoECs, HPMVECs, and EAhy926 were treated with L243 for 3 h (*N* ≥ 3). Data are presented as mean ± SEM. ***P < 0.001. NT not treated, IsoCtrl isotype control

To upregulate levels of endothelial HLA II antigens, which are not constitutively expressed in cell cultures of human ECs, human umbilical vein endothelial cells (HUVECs) were treated with interferon gamma (IFN-γ) for up to 4 days. Expression of HLA-DR was upregulated by IFN-γ in a time-dependent manner (Figure S1). Exposure of IFN-γ-stimulated ECs to the HLA-DR mAb L243 for 3 h induced cell death as determined by annexin V/propidium iodide (PI) staining (Fig. 1a). Levels of cell death by L243 in HUVECS were markedly lower compared to those by treatment with the combination of cycloheximide (CHX) and tumor necrosis factor (TNF)-α (Fig. 1a). Because L243 has recently been shown to cause cell proliferation in cell cultures of human ECs after 48 h^21^, levels of cell death were also determined in long-term cell cultures of human ECs. After 48 h, levels of PI-positive cells were similar in L243-treated and control cells (Fig. 1b) suggesting that HLA-DR-mediated cell death is an early event in a portion of ECs, but is not apparent in cell cultures of HUVECs after extended periods of time. Cell death in L243-treated ECs after treatment for 3 h was also confirmed by LIVE/DEAD staining (Fig. 1c). In the following, annexin V positivity was utilized as a surrogate indicator for cellular damage of ECs to explore the mechanisms that may cause cell death in our experimental setting. In time response studies treatment with L243 induced annexin V positivity in IFN-γ-stimulated ECs, which was proportional to levels of HLA-DR expression (Fig. 1d, Figure S1). Moreover, the effect of the HLA II mAb L243 in ECs was also compared with that of mAbs directed against other distinct HLA epitopes. Notably, the pan HLA I mAb W6/32 did not induce annexin V positivity despite high levels of binding to ECs. By contrast, the independent HLA II mAb TÜ39 markedly increased annexin V staining in ECs (Fig. 1e). Binding of L243 and annexin V positivity occurred in a dose-dependent manner reaching a maximum at a concentration of 5 μg/ml, which indicated saturation of L243-mediated cell death (Fig. 1f). Induction of annexin V positivity by L243 was also observed in various types of primary ECs, including human dermal microvascular endothelial cells (HDMVECs), human aortic endothelial cells (HAoECs), and human pulmonary microvascular endothelial cells (HPMVECs), but not in the EC line EA.hy926 (Fig. 1g, Figure S2). The data suggest that HLA II antibodies induce cell death in primary human ECs.

**Fig. 2:**
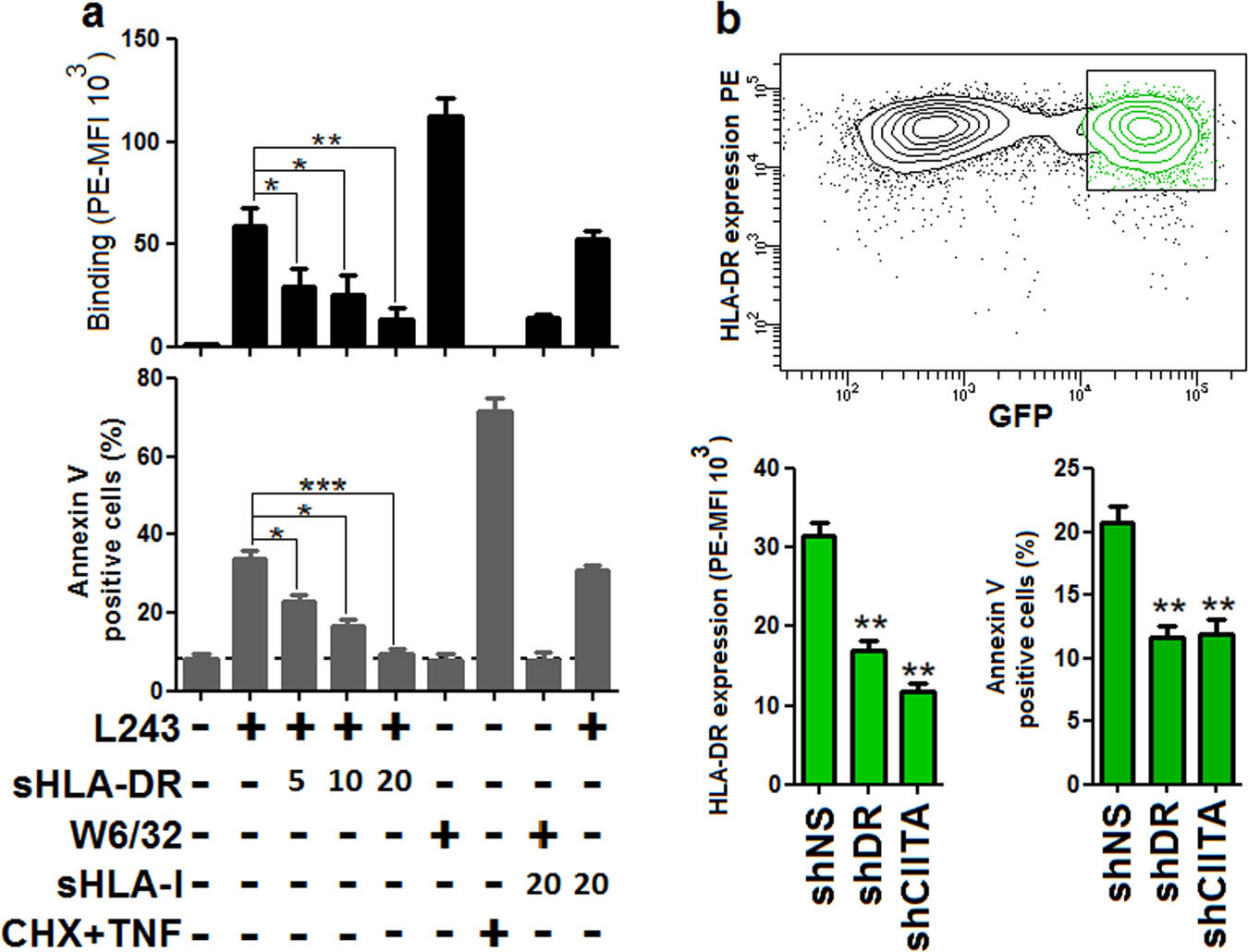
HLA-DR mAb L243 mediates cell death via specific ligation to HLA-DR in human ECs. **a** The HLA-DR mAb L243 (5 µg/ml) was incubated with the indicated concentrations (µg/ml) of sHLA-DR or sHLA I proteins for 1 h at 37 °C before treatment of HUVECs for 3 h (*N* = 3). Cells were subjected to flow cytometry to determine antibody binding and levels of annexin V positivity. Data are presented as mean ± SEM. *P < 0.05, **P < 0.01, ***P < 0.001. **b** HUVECs were transduced with lentiviral vectors encoding for GFP and control of a non-specific short hairpin RNA (shNS), sequences targeting HLA-DR (shDR) or class II transactivator (shCIITA). After treatment with IFN-γ for 3 days, cells were incubated for 3 h with L243. Cells were subjected to flow cytometry to determine HLA-DR antibody binding and cytotoxicity in GFP-positive cells population. Data are presented as mean ± SEM (*N* = 3). **P < 0.01. GFP green fluorescent protein

To assess whether L243-dependent cytotoxicity was mediated via specific binding to endothelial HLA-DR molecules, L243 was preincubated with soluble recombinant HLA (sHLA)-DR and for a comparison with sHLA I protein. Pre-incubation with sHLA-DR, but not with HLA I protein, reduced binding to and annexin V positivity of the mAb L243 in a dose-dependent manner (Fig. 2a). To further substantiate these findings, HLA-DR expression was downregulated via lentiviral knockdown targeting HLA-DR and the master HLA II regulator class II transactivator (CIITA) in ECs. Downregulation of HLA-DR in HLA-DR- and CIITA-targeted cells was corresponding with reduced levels of annexin V positivity in comparison with control cells (Fig. 2b, Figure S3). Notably, HLA I expression levels were not affected by knockdown of HLA-DR (Figure S4). Collectively, the data indicate that cell death by HLA-DR ligation is specific in human ECs and also rule out off-target effects of HLA II antibodies.

### Induction of cell death by native allele-specific HLA-DR antibodies from allosera in ECs

**Fig. 3:**
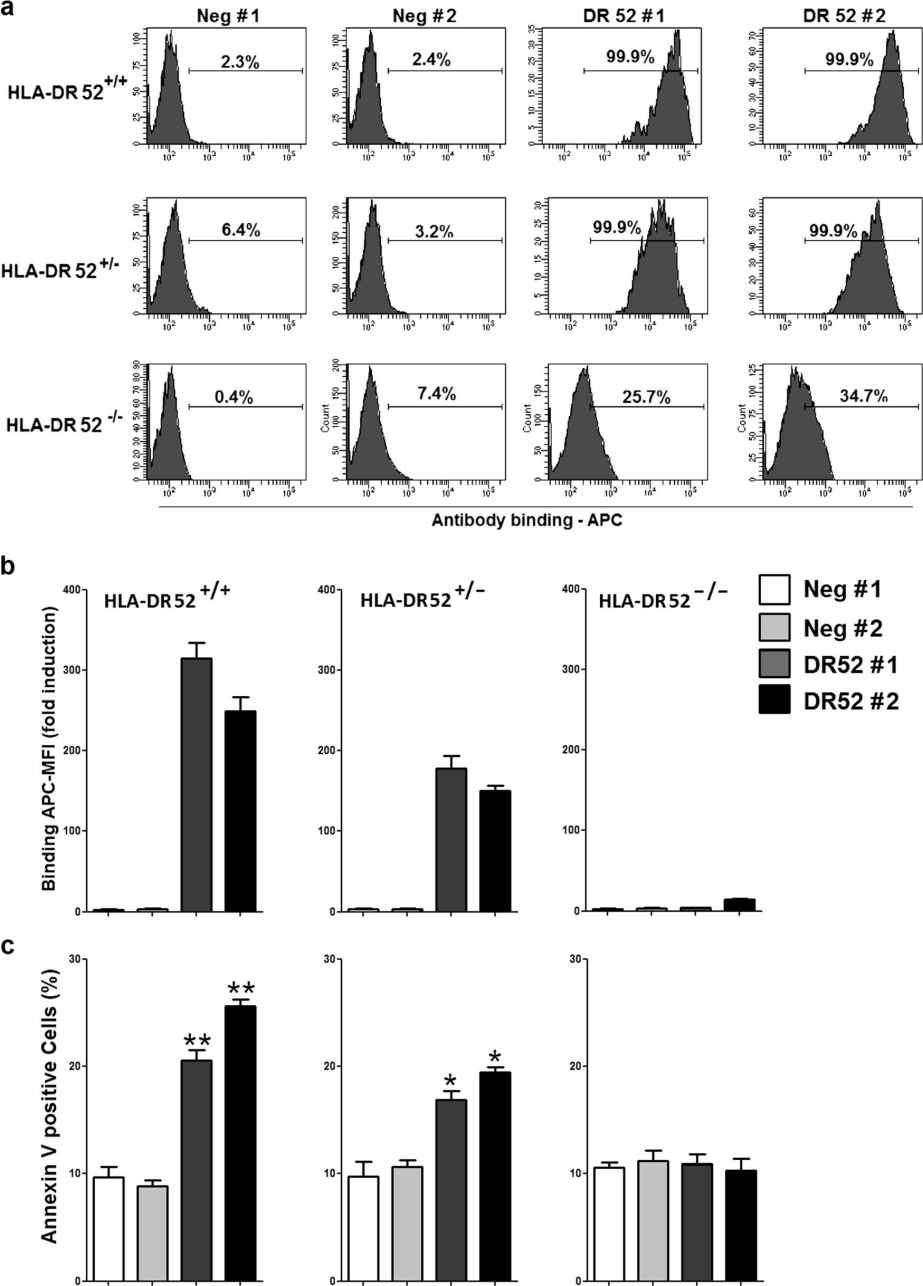
Native allele-specific HLA-DR alloantibodies from human allosera induce cell death in ECs. After pretreatment with IFN-γ for 4 days, HLA-typed HUVECs from three individuals (see Materials and methods) were incubated with control sera (Neg #1 and #2) and HLA-DR52-positive sera from two allo-immunized patients (DR52 #1 and #2). Cells were incubated with the indicated sera for 24 h, after which cells were analyzed for antibody binding (**a**, **b**) and annexin V positivity (**c**). **a–c** Data show a representative flow cytometry histogram and statistical results mean ± SEM from three independent experiments. *P < 0.05; **P < 0.01 significant differences HLA-DR52-positive sera versus control

MAbs against HLA-DR have previously been shown to induce necrotic cell death in various cell types, including leukemia and lymphoma cells^23,32,33^. To determine whether native HLA-DR antibodies might also mediate cell death in ECs, we utilized allosera with allele-specific HLA-DR52 antibodies (Table S1), in which complement activity was inactivated by heat. In HLA-typed HUVECs from independent donors, sera with HLA-DR52 alloantibodies bound to and induced annexin V staining in HLA-DR52-positive HUVECs (Fig. 3). This effect was dose-dependent, because HLA-DR52-positive sera caused higher levels of annexin positivity in homozygous HLA-DR52 (HLA-DR52^+/+^) as compared with heterozygous HLA-DR52^+/−^ ECs (Fig. 3). By contrast, HLA-DR52-negative (HLA-DR52^−/−^) HUVECs neither exhibited binding nor annexin V positivity after treatment with sera containing specific HLA-DR52 antibodies. The findings indicate that native allele-specific HLA-DR antibodies from allosera cause cell death in human ECs.

### HLA-DR antibody-mediated cytotoxicity in ECs is independent of apoptosis and necroptosis

**Fig. 4:**
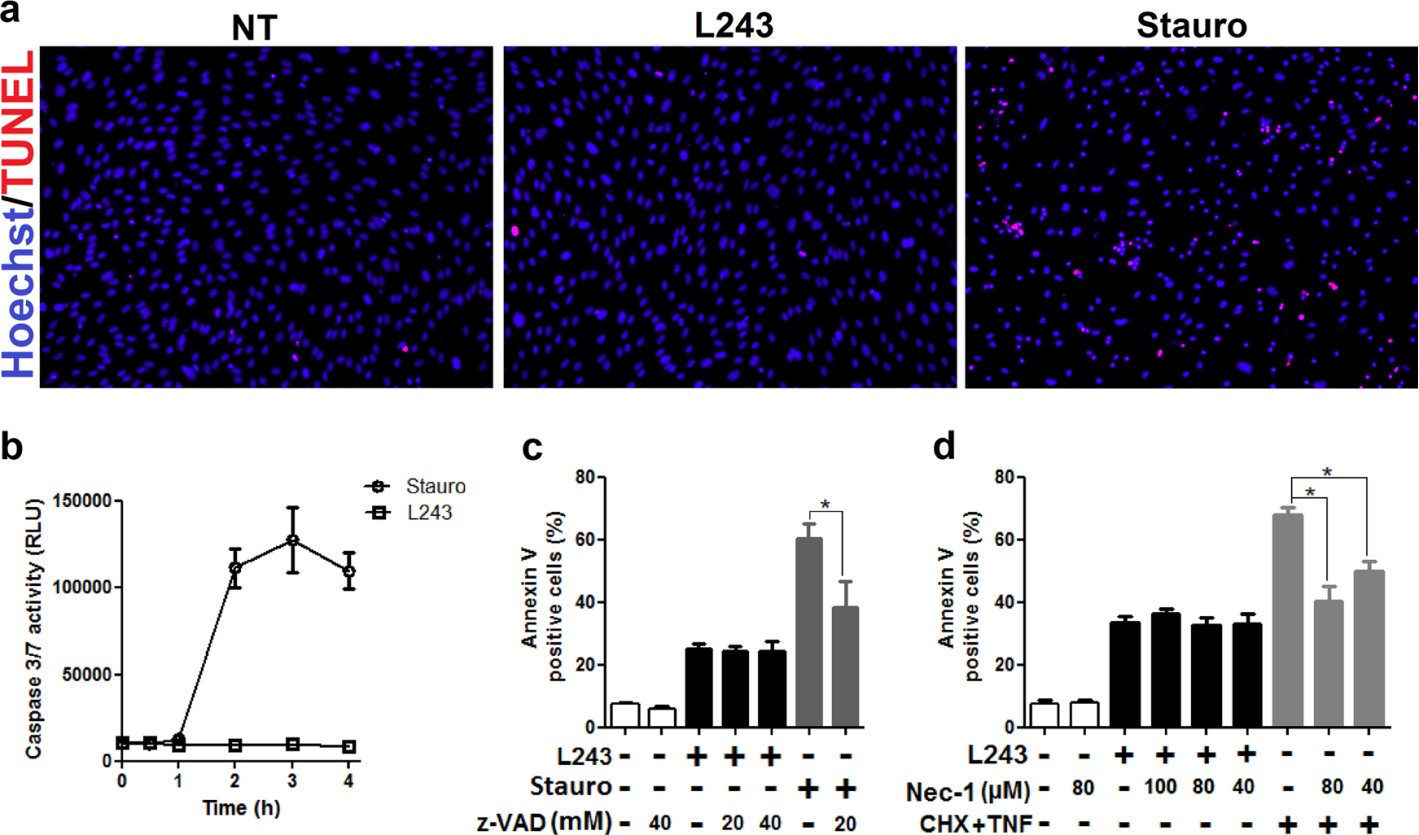
HLA-DR-dependent cell death in ECs is not mediated via apoptosis or necroptosis. **a, b** After pretreatment with IFN-γ for 4 days, HUVECs were incubated with L243 (□) or 50 nM Stauro (o) for 4 h (*N* = 3). **a** Cells were subjected to the TUNEL assay as described in Materials and methods. **b** Activities of caspases were assessed with the Caspase-Glo® 3/7 assay system in a luminometer. **c** After treatment with zVAD for 30 min in order to block apoptosis, HUVECs were treated with L243 or stauro (50 nM) for up to 4 h (*N* = 3). **d** After pretreatment with Nec-1 for 30 min to block necroptosis, HUVECs were incubated with L243 or CHX plus TNF for 4 h (*N* = 3). Data are presented as mean ± SEM. *P < 0.05. RLU relative light units, Stauro staurosporine

To examine whether HLA-DR antibody ligation may cause apoptosis in ECs, we determined DNA fragmentation by TUNEL assay and activities of caspases 3 and 7 as indicators of this pathway in L243-treated cells. Both, TUNEL assay and caspase 3 and 7 activities were not affected by L243, but were markedly upregulated in ECs treated with staurosporine (Stauro), which is a prototypical inducer of apoptosis in ECs (Fig. 4a, b). Moreover, the effect of the pan-caspase inhibitor z-VAD-fmk (zVAD) was determined in L243-treated ECs, which significantly reduced annexin V positivity caused by treatment with Stauro, but not with L243 (Fig. 4c). We also determined the effect of the pharmacological necroptosis inhibitor necrostatin-1 (Nec-1) in ECs. Nec-1 had no effect on HLA-DR-induced annexin V positivity, but inhibited that by TNF-α plus CHX (Fig. 4d). The potential role of ferroptosis in this L243-mediated pathway could not be determined in further detail (eg., via utilization of pharmacological inhibitors), because the prototypical ferroptosis inducers erastin and RSL3 did not induce cell death in cell cultures of human ECs (data not shown). Taken together, the data indicate that cell death induced by HLA-DR antibody ligation in ECs is mediated via a necrotic pathway independent of apoptosis and necroptosis.

### Increased generation of reactive oxygen species (ROS) and altered mitochondria membrane potential in HLA-DR antibody-treated ECs

**Fig. 5:**
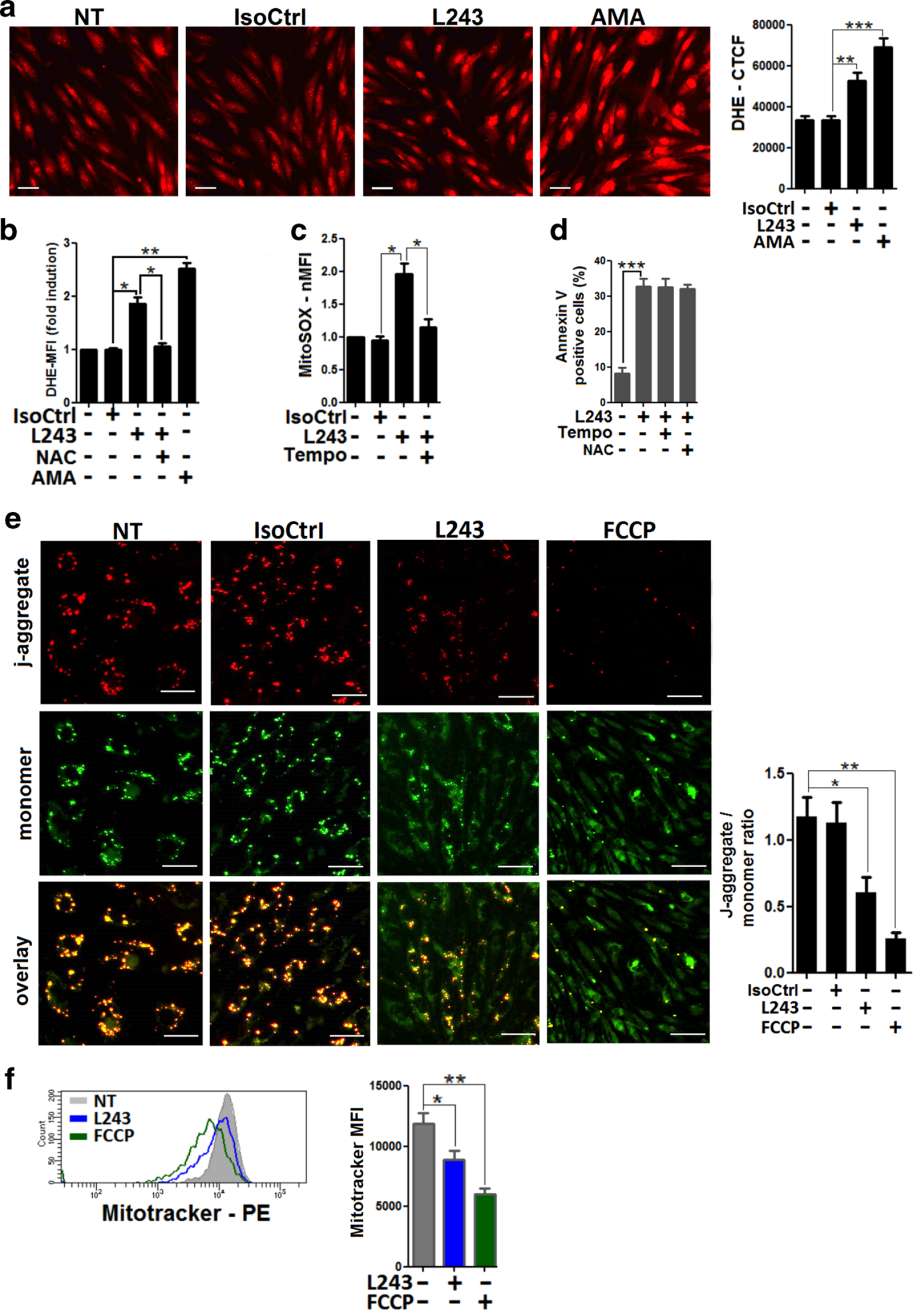
Increased cellular ROS and loss of mitochondrial membrane potential in HLA-DR antibody-treated ECs. HUVECs were pretreated with IFN-γ for 4 days. **a** DHE-preloaded HUVECs were treated for 1 h with L243 or AMA. A representative image from three independent experiments is shown (scale bars = 60 µm). Bars show means ± SEM from DHE-corrected total cell fluorescence. **P < 0.01; ***P < 0.001. **b** After pretreatment with NAC, HUVECs were exposed to L243 for 1 h in the presence of DHE and were subjected to flow cytometry. Bars represent mean ± SEM of fold induction of DHE-MFI levels. *P < 0.05; **P < 0.01. **c** After pretreatment of MitoSOX-exposed HUVECs with mito-Tempo, L243 was added for 1 h as described in Materials and methods. Bars represent mean ± SEM of fold induction (MFI), as determined by flow cytometry from three independent experiments. *P < 0.05. **d** After pretreatment of HUVECs with NAC and mito-Tempo, L243 was added for 3 h (*N* = 4). Cells were assessed for annexin V positivity by flow cytometry. Data are presented as mean ± SEM. **e** HUVECs were treated for 1 h with the indicated mAbs or FCCP and stained with JC-1 as described in Materials and methods. A representative image of three independent experiments is shown (scale bars = 30 µm). Bars represent the ratio of red j-aggregate to green monomer as mean ± SEM from three independent experiments. *P < 0.05; **P < 0.01. **f** HUVECs were treated with L243 or FCCP for 3 h, as indicated. Cells were then stained with Mitotracker Red. Data show a representative flow cytometry histogram. Bars show Mitotracker Red MFI means ± SEM from three independent experiments. *P < 0.05; **P < 0.01. AMA antimycin A, MFI mean fluorescence intensity, NAC N-acetyl-L-cysteine

Mitochondria are critically involved in signal transduction by numerous environmental stimuli and control cellular life and death in the endothelium^34–36^. Because redox-dependent mechanisms are important for mitochondria-dependent signaling in ECs^35^, the generation of ROS was determined via dihydroethidium (DHE) staining. Upon treatment with L243, high levels of intracellular ROS were detected by fluorescence microscopy (Fig. 5a) and by flow cytometry (Fig. 5b). Levels of ROS in L243-treated ECs were lower compared to those treated with the respiratory chain inhibitor antimycin A (AMA), which is a prototypical inducer of cellular ROS. Moreover, L243-mediated ROS production was blocked by treatment with the antioxidant radical scavenger N-acetyl-L-cysteine (NAC) (Fig. 5b). To explore the potential role of mitochondria as a source of endothelial ROS, MitoSOX staining was applied in flow cytometry studies. Clearly, levels of mitochondrial superoxide were markedly upregulated in L243-treated ECs (Fig. 5c). The role of mitochondria for endothelial production of ROS was also supported by the finding that pretreatment with the superoxide dismutase inhibitor mito-TEMPO largely reduced levels of superoxide (Fig. 5c). However, pretreatment with these antioxidant compounds did not reduce cell death in ECs after treatment with L243 (Fig. 5d). To explore whether enhanced generation of ROS in ECs was associated with mitochondrial stress, alterations of the mitochondrial membrane potential were determined by studies with JC-1. Mitochondrial membrane potential in ECs was downregulated after L243 treatment to a slightly lower extent in comparison to that observed for carbonyl cyanide 4-(trifluoromethoxy) phenylhydrazone (FCCP), which is a known inducer of mitochondrial stress (Fig. 5e). Finally, loss of mitochondrial membrane potential was also determined by Mitotracker Red staining in L243-treated ECs (Fig. 5f). Collectively, the data indicate that HLA-DR antibody ligation causes mitochondrial membrane disruption and increased generation of ROS in ECs.

### HLA-DR antibody ligation induces lysosomal activation, LMP, and rupture in ECs

**Fig. 6:**
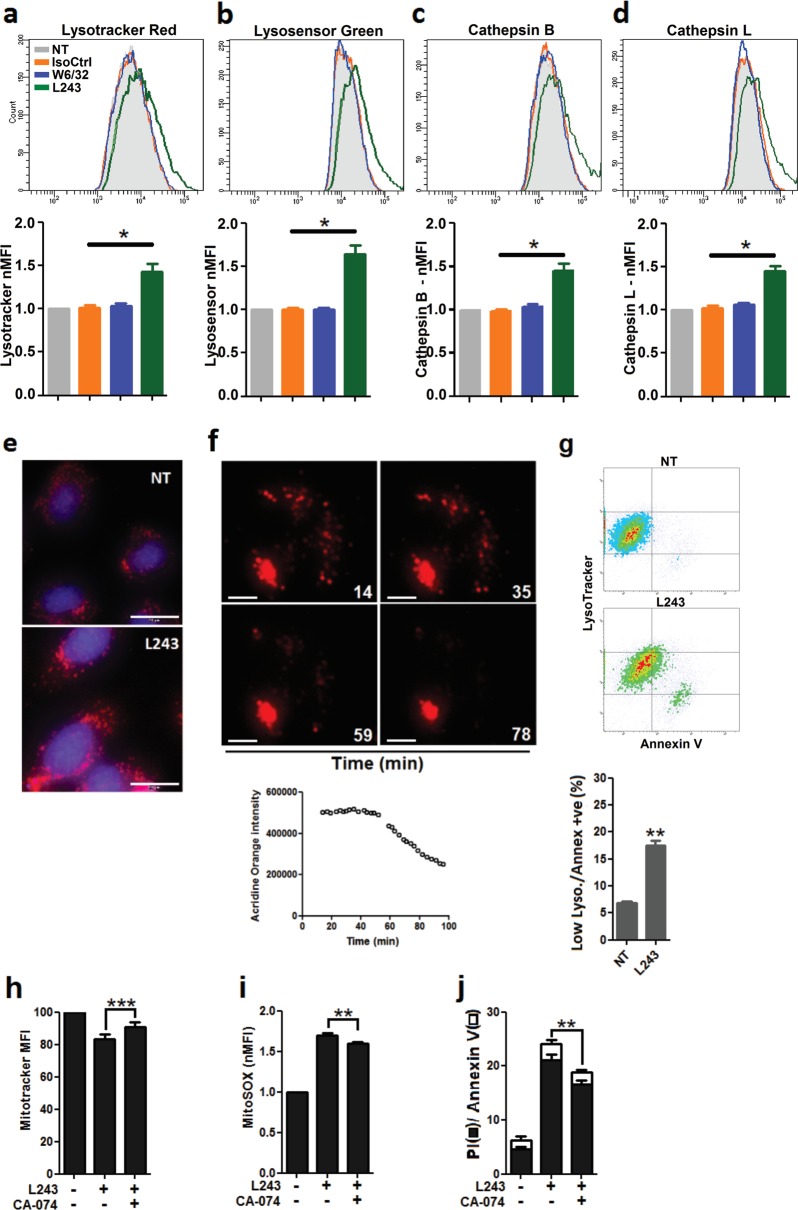
Lysosomal hyperactivation and LMP in ECs treated with HLA-DR antibody. **a**–**d** After pretreatment with IFN-γ for 4 days, HUVECs were incubated with the indicated antibodies for 3 h. Cells were then stained with (**a**) Lysotracker Red to measure lysosomal appendency, (**b**) Lysosensor Green to measure lysosomal acidity, and (**c, d**) Magic Red for cathepsins B and L activity, respectively (*N* = 3). **a**–**d** MFI of stains was measured by flow cytometry. Data are presented as mean ± SEM. *P < 0.05. **e** HUVECs were treated with L243 for 3 h and then stained with Magic Red to determine cathepsin L activity. A representative image of three independent experiments is shown (Bars = 20 µm). **f** After pretreatment with IFN-γ for 4 days, HUVECs were loaded with AO (10 µg/ml), after which cells were treated with L243 and microscopic images were taken. Data show a representative image from three independent experiments. The line chart shows the AO red fluorescence intensity from time-lapse recording for up to 100 min. **g** HUVECs were treated with L243 for 3 h and stained with Lysotracker Red and annexin V FITC (*N* = 3). Cells were subjected to flow cytometry to determine the relationship between lysosomal volume and cell death. Data are presented as mean ± SEM. *P < 0.05, **P < 0.01. **h–j** After pretreatment with CA-074 for 30 min in order to block cathepsin B activity, HUVECs were incubated with L243 for 3 h (*N* = 4). **h, i** For evaluation of mitochondrial alterations, adherent cells were loaded with Mitotracker and MitoSOX before analysis. **j** Cytotoxicity was assessed by flow cytometry with PI (■) and annexin V (□). **P < 0.01 significant differences CA-074 plus L243 versus L243 alone for both ■ and □. IsoCtrl isotype control, MFI mean fluorescence intensity, NT non-treated, CA-074 CA-074 Me

Mitochondrial stress can be a downstream consequence of functional lysosomal alterations, such as LMP^37,38^ (for a review see ref. ^39^). To explore the potential role of lysosomes in HLA-DR antibody-mediated endothelial damage, we determined lysosomal mass and acidity along with activities of cathepsins B and L in cell cultures of HUVECs. Staining with Lysotracker Red revealed an increase of total lysosome abundance after treatment with L243 (Fig. 6a), which was accompanied by increased lysosomal acidity as indicated by staining with Lysosensor Green (Fig. 6b). Both parameters correlated with upregulated activity of the key lysosomal cell death executors cathepsin B and L suggesting lysosomal hyperactivation in response to L243 (Fig. 6c, d). This was also confirmed by increased cathepsin activity in immunofluorescence staining (Fig. 6e). As increased cathepsin activity in lysosomes is associated with LMP^39,40^, the integrity of lysosomes was also assessed with acridine orange (AO) staining^41^. In the early phase of L243 treatment, ECs exhibited a distinct AO red pattern as signature of intact lysosomes. AO intensity, however, was markedly decreased after 50 min indicating the presence of LMP (Fig. 6f). To correlate changes of the lysosomal volume with cell death, ECs were labeled with Lysotracker Red and then stained with annexin V. The increase in annexin V positivity was associated with loss of lysosomal staining indicating lysosomal rupture (Fig. 6g). To determine whether lysosomal rupture and release of cathepsin-B into the cytosol may lead to cell death, ECs were pretreated with the cathepsin B inhibitor CA-074 Me (CA-074) before stimulation with mAb L243. CA-074 reduced mitochondrial damage and cell death in ECs after L243 treatment (Fig. 6h–j). Overall, these results show that HLA-DR antibody ligation causes lysosomal hyperactivation, LMP, and release of cathepsins into the cytosol.

### Reorganization of the cytoskeleton is involved in HLA-DR-mediated cell death in ECs

**Fig. 7:**
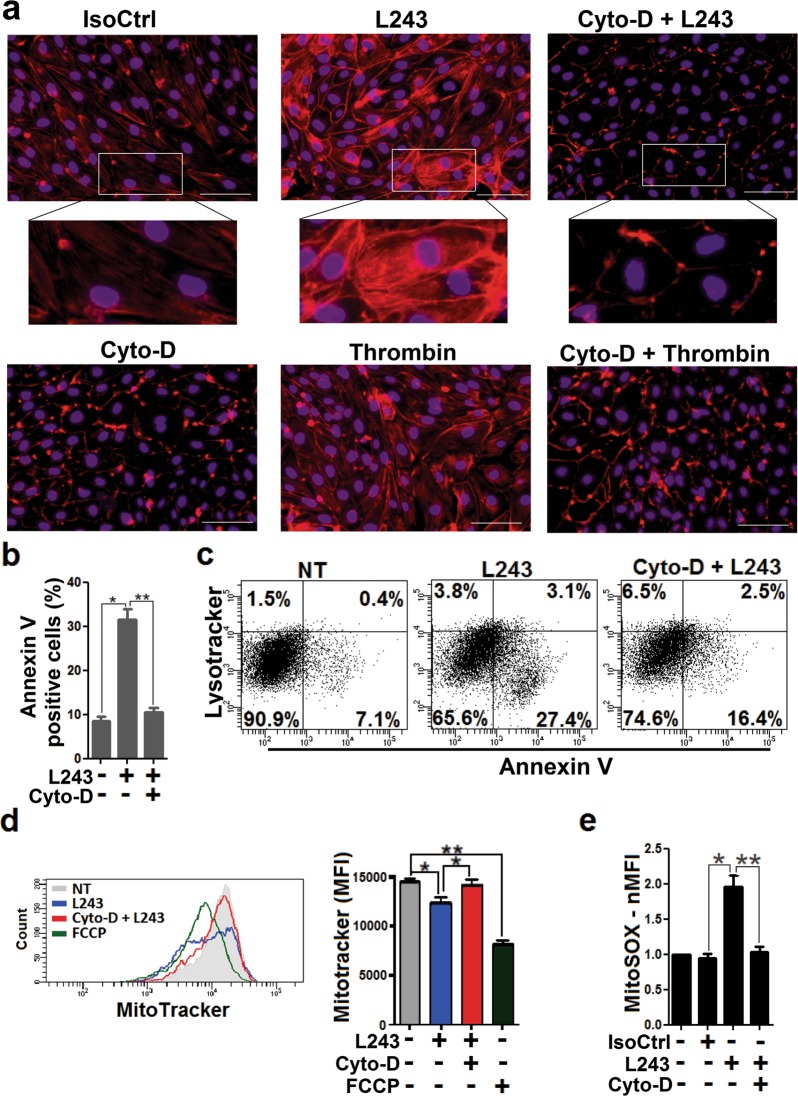
Stress fiber formation in ECs treated with HLA-DR antibody and effect of Cyto-D. **a** After pretreatment with IFN-γ for 4 days, HUVECs were treated with isotype control antibody (IsoCtrl), mAb L243, or thrombin in the absence or presence of Cyto-D (100 nM) for 3 h, as indicated. Cells were then incubated with Texas Red Phalloidin for staining of F-actin stress fiber formation and DAPI for staining of nuclei (*N* = 3). Representative images (Bars = 100 µm) with insets of higher resolution are shown. **b** HUVECs were treated with L243 in the absence or presence of Cyto-D (100 nM) for 3 h (*N* = 3), as indicated. Cells were assessed for annexin V positivity by flow cytometry. Data are presented as mean ± SEM. *P < 0.05, **P < 0.01, ***P < 0.001. **c** HUVECs were treated with L243 for 3 h and stained with Lysotracker Red and annexin V FITC (*N* = 3). Cells were subjected to flow cytometry to determine the relationship between lysosomal volume and cell death. A representative flow cytometry dot plot from three independent experiments is shown. **d** HUVECs were treated with L243 or FCCP in the absence or presence of Cyto-D for 3 h, as indicated. Cells were then stained with Mitotracker Red. Data show a representative flow cytometry histogram and statistical results mean ± SEM from three independent experiments. **e** After pretreatment of MitoSOX-exposed HUVECs with Cyto-D, L243 was added for 1 h as described in Materials and methods. Bars represent mean ± SEM of fold induction (MFI), as determined by flow cytometry from three independent experiments. *P < 0.05, **P < 0.01. IsoCtrl isotype control, Cyto-D cytochalasin D

LMP-dependent cell death via ligation of the mAb L243 in leukemia cells and signal transduction by this mAb in a lymphoma cell line have been associated with remodeling of the actin cytoskeleton^23,42^. To explore the potential role of cytoskeleton regulation for HLA-DR-dependent intracellular effects in human ECs, we determined the effects of L243 on F-actin polymerization. L243 induced the formation of cytoplasmic F actin stress fibers in ECs, which was similar to that observed for treatment with thrombin. Remarkably, pretreatment with the potent actin polymerization inhibitor cytochalasin D (Cyto-D) attenuated F actin stress fiber formation by L243 (Fig. 7a, Figure S5). To find out whether reorganization of the cytoskeleton could be involved in HLA-DR-mediated cytotoxicity of ECs, we also determined the effect of Cyto-D on annexin V positivity in L243-treated cells. L243-mediated annexin V staining was abrogated by Cyto-D (Fig. 7b), and a similar effect was also observed for a second actin polymerization inhibitor, latrunculin B (Lat-B) (data not shown). Since cytoskeleton remodeling is critically controlled by Rho GTPases, ECs were also pretreated with the cell-permeable Rho GTPase inhibitor CT04. CT04 reduced actin stress fiber formation and cytotoxicity by L243 (Figure S6). To investigate whether L243-dependent cytoskeleton regulation would cause downstream alterations in mitochondria and lysosomes (Figs. 5 and 6), the effects of Cyto-D were also determined in these endothelial organelles. Treatment with Cyto-D reduced L243-mediated lysosome rupture as indicated by Lysotracker Red staining (Fig. 7c). Moreover, this compound blocked loss of mitochondrial membrane potential and mitochondrial superoxide production in L243-treated ECs (Fig. 7d, e). In conclusion, the data indicate that cytoskeleton remodeling are critically involved in HLA-DR-dependent cell death of ECs.

## Discussion

AMR plays a major role for long-term graft survival after solid organ transplantation^3–6^. The endothelium of transplanted organs is critically involved in the pathogenesis of AMR, because ligation of HLA antibodies to ECs mediates transplant rejection via complement-dependent and -independent effects^8,11,12,15^. In the current study, a cell culture model of HLA-DR antibody-dependent stimulation was utilized to investigate the effects of HLA II antibodies in primary human ECs. It is demonstrated that endothelial HLA-DR ligation induced complement-independent necrotic cell death via a pathway that is mediated by reorganization of the cytoskeleton and LMP.

### Induction of necrotic cell death by HLA-DR antibody ligation in ECs

HLA-DR antibody ligation induces necrotic cell death in primary human ECs (Fig. 1), the specificity of which is supported by the following lines of evidence. First, the pan HLA I mAb W6/32 did not cause cell death despite high levels of binding to IFN-γ-stimulated ECs (Fig. 1e). Second, in addition to the HLA-DR mAb L243, the independent HLA II mAb TÜ39 induced cytotoxicity (Fig. 1e). Third, HLA-DR-dependent EC death by L243 was blocked by pre-incubation with specific recombinant HLA-DR protein (Fig. 2a). Finally, knockdown of HLA-DR via lentiviral transduction of ECs prevented cytotoxicity by mAb L243 (Fig. 2b). Remarkably, HLA-DR antibody-mediated necrotic cell death was not only observed for mAbs (Fig. 1, Fig. 2), but also for native allele-specific HLA antibodies from allosera (Fig. 3). Although complement-dependent effects of HLA antibodies have been considered to be the major cause of AMR for many years, the role of complement-independent effects in this condition has been established more recently and is supported by a large body of experimental evidence (for reviews see refs. ^8,11,12^). For example, HLA I antibodies activate intracellular signaling cascades that induce inflammatory activation of ECs^17,43^. In contrast, the functional role of HLA II antibody ligation with ECs has remained largely unknown. In the few reports on this topic, HLA-DR-dependent stimulation of ECs has been associated with activation of protein kinase C^44^, upregulation of IL-6 secretion^20^ or cell proliferation and migration^21^. The apparent discrepancy of our current findings to these earlier reports may be explained by differences in the utilized cell culture models and experimental conditions. For example, lower expression levels of endothelial HLA-DR due to various concentrations and lengths of exposure time to IFN-γ and different cell types of ECs have been reported in these earlier studies^20,44^. Notably, in our experimental setting HLA-DR antibodies induce cell death only in a portion of ECs in short-term cell cultures (3 h), but not long-term cell cultures (48 h) (Fig. 1b), suggesting that not all cells are equally susceptible to HLA-DR-mediated cytotoxicity. Time-dependent differences may also explain why the HLA-DR-specific mAb L243, which is applied in most experiments of the present study, may also cause cell proliferation in human ECs in long-term cell cultures^21^. Thus, it is interesting to note that increased proliferation has been demonstrated for ECs after initial resistance to apoptosis in an experimental rat model of angioproliferative pulmonary hypertension^45^. It is also conceivable that HLA-DR antibody ligation may cause different, seemingly contradictory effects in human ECs. This has been demonstrated for members of the TNF-family, which can cause opposing pro-survival and pro-death effects^46^. Moreover, our current findings agree with earlier reports demonstrating induction of necrotic cell death by HLA-DR mAb L243 in various types of cells, including B cells, dendritic cells, and lymphoma cells^22–24,47,48^. Finally, it is noteworthy that other anti-endothelial antibodies such as anti-NS1, which is critical for the pathogenesis of the dengue hemorrhagic syndrome, or so-called non-HLA antibodies from immunized transplant patients can also cause complement-independent cell death in ECs^49,50^.

### HLA-DR-dependent cell death is mediated via a LMP-dependent pathway involving reorganization of the cytoskeleton in human ECs

**Fig. 8:**
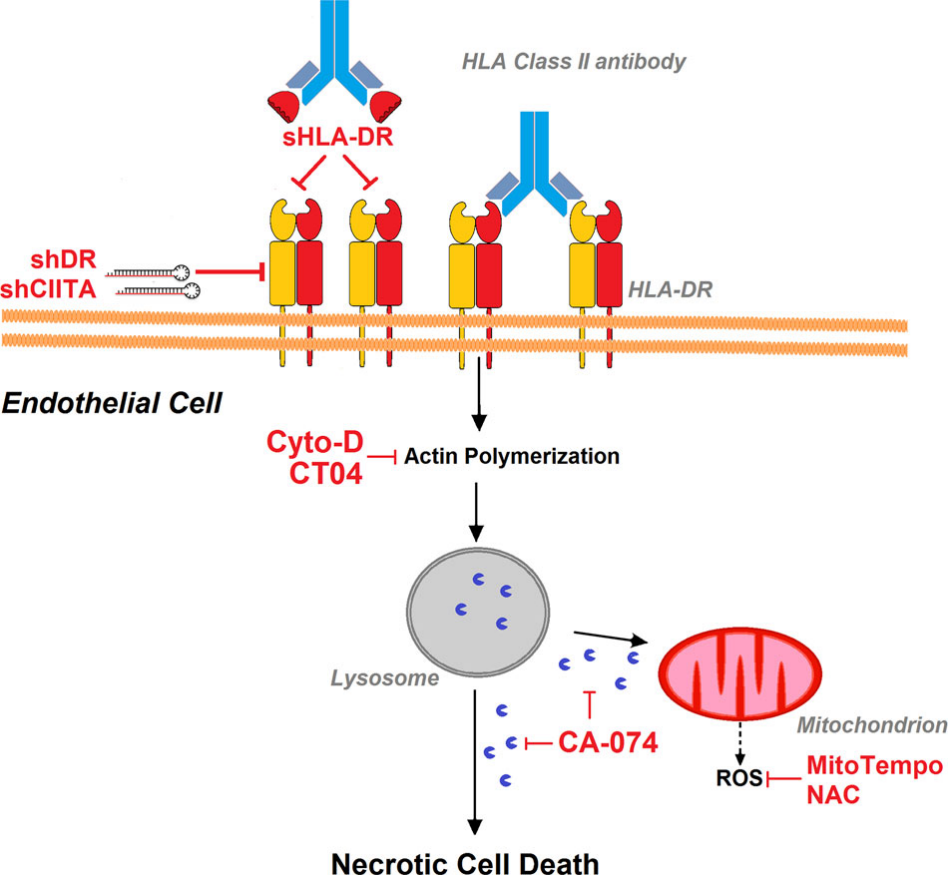
HLA-DR-induced necrotic cell death in human ECs. Schematic diagram on the proposed pathway that mediates HLA-DR ligation-dependent cell death in ECs

HLA II antibody-dependent induction of cell death in ECs is mediated via a LMP-dependent pathway that is associated with upstream F actin cytoskeleton remodeling and lead to mitochondrial stress (Fig. 8). Ligation of HLA-DR mAb L243 to ECs led to lysosomal hyperactivation, LMP, and rupture of these organelles, suggesting that lysosomal destabilization plays a major role for this cell death pathway (Fig. 6). Hyperactivity of lysosomes, which is indicated by increased cathepsin activity, lysosomal mass, and acidity (Fig. 6), has previously also been shown to reduce lysosomal membrane stability in the microenvironment of cancer cells^39,40,51^. HLA-DR-mediated LMP was downstream of F actin stress fiber formation in ECs (Fig. 7c), which is in agreement with findings of previous reports^23,39,40^. Moreover, HLA-DR-dependent cytoskeleton alterations and LMP in ECs appear to be causally linked to mitochondrial stress, which is revealed by loss of mitochondrial membrane potential and generation of mitochondrial ROS (Figs. 6 and 7). It is well known that LMP and/ or lysosomal disruption can be associated with mitochondrial stress^37,38,52^. Finally, generation of mitochondrial ROS in response to HLA II antibodies, which do not directly cause cell death (Fig. 5d), may be involved in an amplifying loop between lysosomes and mitochondria inducing regulated necrosis (for reviews see refs. ^39,40^). The potential role of LMP in regulated necrosis has been subject of on-going discussions because most previous data suggested LMP as a downstream mechanism in almost any signaling pathway of regulated cell death, including late apoptosis/secondary necrosis^25^. Compelling evidence on its functional significance has also come from studies in lysosomal storage diseases and cancer^51,53,54^. Clearly, the data of the present study add to the growing body of experimental evidence, which supports a major role of LMP as a specific pathway of regulated necrosis.

### Clinical and translational aspects of HLA-DR mediated cell death in ECs

AMR is a major limiting factor for graft survival after transplantation of solid organs, including the kidney, heart, and lung^7,8^. Interactions of HLA antibodies, in particular those of DSAs with the allograft endothelium play a critical role in transplant rejection^11^. Therefore, the herein demonstrated pathway not only adds to previously described modes of HLA antibody-mediated regulatory mechanisms of the endothelium, but also gives novel insights into the pathogenesis of AMR. In particular, as necrotic cell death is associated with release of intracellular content and pro-inflammatory cytokines, collectively referred to as DAMPs, HLA-DR-mediated release of such compounds during necrotic cell death of ECs may link AMR to necroinflammation^31,55^. Due to the limited clinical success of current therapeutic regimens in AMR, these findings may enable the development of novel therapeutic approaches for this complex disorder.

In conclusion, it has been demonstrated that ligation of HLA-DR antibodies with ECs induces necrotic cell death via a complement-independent pathway that involves remodeling of the cytoskeleton and LMP. This pathway may not only play a critical role in the pathogenesis of AMR, but may also serve as a therapeutic target for treatment of transplant rejection in solid organ transplantation.

## Materials and methods

### Antibodies and chemicals

HUVECs, HPMVECs, and HDMVECs were from PromoCell (Heidelberg, Germany) and HAoECs from Lonza (Cologne, Germany). Recombinant human IFN-γ and TNF-α were from PeproTech (Hamburg, Germany). Isotype control antibody was from AbD Serotec (Düsseldorf, Germany). Hybridoma cell lines for the mAbs W6/32 (HB-95) and L243 (HB-55) were purchased from American Type Culture Collection, and hybridoma clone for mAb TÜ39 was from Professor Andreas Ziegler (Charité, Berlin). MAbs were purified by protein A-agarose affinity chromatography. For a comparison, mAb L243 was also purchased from Bio-Rad (Düsseldorf, Germany) and Biolegend (Koblenz, Germany). The sHLA class I and II proteins were purchased from imusyn (Hannover, Germany). Caspase-Glo^®^ 3/7 assay, and zVAD were from Promega (Mannheim, Germany). CHX, Nec-1, FCCP, Stauro, DHE, NAC, AMA, mito-TEMPO, erastin, Cyto-D, and Lat-B were from Sigma-Aldrich (Steinheim, Germany). RSL3 was from Cayman Chemical Company (Ann Arbor, USA). Annexin V apoptosis detection kit with PI assay, PE-conjugated goat anti-mouse Fc, and APC-conjugated goat anti-human IgG antibodies were from Biolegend (Koblenz, Germany). LIVE/DEAD cell viability assay, LysoTracker™ Red DND-99, LysoSensor™ Green DND-189, MitoTracker™ Red CMXRos and MitoSOX™ Red staining were from Thermo Fisher Scientific (Darmstadt, Germany). JC-1 and Cell Meter TUNEL Apoptosis Assay Kit were from Biomol (Hamburg, Germany). Magic red cathepsin B and L assay kits and AO were from ImmunoChemistry Technologies (Bloomington, MN, USA). Cathepsin B inhibitor CA-074 and the cell-permeable Rho I inhibitor CT04 were from Enzo Life Sciences (Lörrach, Germany) and Biozol Diagnostica (Eching, Germany), respectively. Sera from allo-immunized patients or individuals were from the Department of Immunohematology and Blood Transfusion, Leiden University Medical Center (Leiden, Netherlands) or from Hannover Medical School. These studies were approved by a vote of the local ethics committee of Hannover Medical School (Ethics vote No. 1560/2012).

### Cell culture of ECs and treatment with antibodies

Cells were cultured as described previously^56^. In brief, HUVECs (four donors), HAoECs (two donors), HDMVECs and HPMVECs in passages 3 to 8 were cultured in appropriate media from PromoCell (Heidelberg, Germany) with 2% fetal bovine serum (FBS), which was heated to inactivate complement. The EAhy926 cell line was cultured in DMEM with 10% heat-inactivated FBS. Cultured cells at 60–80% confluency were treated with recombinant human IFN-γ (600 IU) in fresh media for 4 days to induce HLA II expression. For studies with HLA-typed cells, HUVECs with different HLA-DR genotypes (HLA-DR52^+/+^: HLA-DRB1*11:01 P/12:01 P [Lot. No. 1022301]), (HLA-DR52^+/−^: HLA-DRB1*11/15 [Lot. No. 1083101]), and (HLA-DR52^−/−^: HLA-DRB1*01:01:01/16:01:01 [Lot. No. 1051004]) were applied. After IFN-γ treatment, ECs in culture media were exposed to 5 µg/ml of mAbs antibodies (stock solution: 1 mg/ml) or 1:4 heat-inactivated human sera from allo-immunized patients at 37 °C for 3 h.

### Flow cytometry for detection of EC binding

ECs were incubated with mAbs or human sera for at least 1 h and recovered in phosphate buffered saline (PBS) with 2% FBS. PE-conjugated goat anti-mouse antibody or APC-conjugated goat anti-human IgG were added to cells for 20 min at room temperature (RT). Cells were washed and antibody binding was assessed with a FACSCanto flow cytometer and FACSDiva software (BD Biosciences, San Jose, CA, USA). The mAb L243 was preincubated with sHLA I and DR proteins at the indicated concentrations at 37 °C for 1 h before incubation with ECs.

### Cell viability

Phosphatidylserine positivity of ECs was assessed by flow cytometry with the “Annexin V apoptosis detection Kit”. Loss of plasma membrane integrity was detected with PI FACS staining or with LIVE/DEAD cell viability assay according to the manufacturers’ instructions, respectively. Moreover, the presence of DNA fragmentation after 4 h was assessed by Cell Meter TUNEL Apoptosis Assay Kit according to the manufacturer’s instructions. In brief, ECs were fixed with paraformaldehyde (4%) for 10 min and permeabilized with 0.3% triton X-100 in PBS for 5 min at RT. Cells were washed and stained with TUNEL assay in PBS for 1 h and nuclei were visualized with Hoechst 33342 (Thermo Fisher Scientific).

### Characterization of HLA alloantibodies in human allosera

Panel reactivity of HLA-DR52-positive sera was determined by complement-dependent cytotoxicity assay and confirmed by single antigen Luminex assay (One Lambda, Canoga Park, CA, USA). Complement activity was heat-inactivated by incubation of allosera at 56 °C in a water bath for 30 min. Binding to ECs was also confirmed by flow cytometry.

### Knockdown of HLA-DR via a lentiviral vector-based approach in ECs

Knockdown of HLA-DR was performed as previously described^57^. In brief, confluent HUVECs in passage 3 were transduced with enhanced green fluorescent protein (GFP) containing lentiviral vectors encoding for sequences targeting HLA-DR (shDR) or CIITA (shCIITA) or a non-specific short hairpin RNA (shNS) as a control, for 24 h. After treatment with IFN-γ for 3 days, cells were examined for HLA-DR expression and antibody-mediated cytotoxicity by flow cytometry in the GFP-positive cell population, representing successfully transduced ECs.

### Staining and quantitation of F-actin stress fiber formation

Stimulated ECs were fixed with paraformaldehyde (4%) for 10 min and permeabilized with 0.3% triton X-100 in PBS for 5 min at RT. Cells were then washed and stained with DAPI (300 nM) and Texas Red™-X Phalloidin (200 nM)(Thermo Fisher Scientific, Darmstadt, Germany) in PBS for 1 h. An Olympus 1 × 81 fluorescence microscope (x10 magnification) was utilized to capture digital images and the fluorescence intensity of a defined field was quantified with ImageJ as described previously^58^. Briefly, the threshold tool in the ImageJ program was used to define an optical density limit that reveals visible F actin fibers and eliminates background fluorescence. Based on the overlay images with DAPI, ~100 cells were taken into account for each field. Using the region of interest (ROI) manager, the fluorescence intensity of the defined field was measured and the following equation was used to calculate corrected total cell fluorescence (CTCF): integrated density - (area of field x mean fluorescence of background). The average CTCF values from three independent experiments are shown as fold induction of stress fiber intensity normalized to the control isotype group to reflect the change with different treatments. Cyto-D (100 nM), Lat-B (100 nM), and CT04 (1 µg/ml) were added to ECs before stimulation to inhibit stress fiber formation, as indicated.

### Lysosomal mass and permeability assays

Lysosomal mass, acidity, and cathepsin activities were assessed with flow cytometry. After stimulation, ECs were stained with LysoTracker Red (75 nM), LysoSensor Green (1 µM), or magic red cathepsin L, B kits (1:1000) according to the manufacturers’ instructions. Cathepsin L activity was also determined using fluorescence microscopy. In brief, HUVECs were stimulated for 3 h and cathepsin L substrate was added to the culture media. To correlate changes in lysosomal mass with loss of lipid composition asymmetry, cells were stained with LysoTracker and annexin as described previously^32^. In intact lysosomes, AO accumulates due to proton trapping to emit red fluorescence. AO-loaded cells manifest reduced red fluorescence after LMP. For detection of LMP by AO ECs were loaded with 10 µg/ml AO for 15 min, after which cells were treated with L243 for 10 min at 37 °C and microscopic images (x40 magnification) were taken at the indicated time points. For quantification of AO-fluorescence, in the ImageJ program, time-lapse images of the selected cell were taken and the fluorescence intensity was measured in the ROI manager and expressed as CTCF using the equation aforementioned. The arbitrary fluorescence values are shown^59^.

### Evaluation of mitochondrial membrane potential

Cells were stained with JC-1 (2 µM) for 20 min and examined with a fluorescence microscope. The mitochondria with normal mitochondrial membrane potential are stained red (due to j-aggregate) and those with decreased mitochondrial membrane potential are stained green (due to monomer stain). Mitochondrial membrane potential was also assessed by flow cytometry with Mitotracker Red CMXROs staining, as previously described^60^.

### Detection of ROS

HUVECs were preloaded with the superoxide probe DHE (5 µM) and cells were stimulated for 1 h with mAbs. In the presence of superoxide, DHE is oxidized and binds to DNA enhancing intracellular fluorescence, which can be visualized under fluorescence microscopy at excitation and emission wavelengths of 540/25 nm and 605/55 nm, respectively. ImageJ was used to process the fluorescent images obtained as described previously^61^. Individual cells were marked and the CTCF was calculated using the aforementioned equation. Approximately, 50 cells from each experiment were taken into account for calculation. The average CTCF values are shown as arbitrary fluorescence values. HUVECs were preloaded with MitoSOX Red (5 µM) to detect mitochondrial ROS and cells were stimulated for 1 h with mAbs. MFI levels were assessed by flow cytometry, as indicated. ECs were pretreated with NAC (5 mM) and MitoTempo (1 mM) for 0.5 and 1 h, respectively.

### Statistical analysis

Statistical analyses were performed by the GraphPad Prism Version 5.02 using paired two-tailed *t* test. Values were considered statistically significant at *P* < 0.05.

## Supplementary information

Supplemental Figures and Tables
