# Supplementary material for: HLA class II antibodies induce necrotic cell death in human endothelial cells via a lysosomal membrane permeabilization-mediated pathway
Source: Cell Death Dis. 2019 Mar 8;10(3):235. doi: 10.1038/s41419-019-1319-5 (PMC6408495; doi:10.1038/s41419-019-1319-5)
Supplement: Supplementary file 1 — Supplemental Figures and Tables [file 41419_2019_1319_MOESM1_ESM.docx]

**SUPPLEMENTARY**

**Supplementary Figures**

**
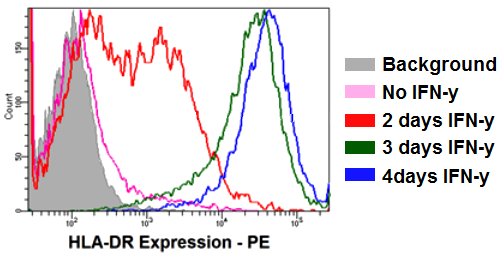
**

**Figure S1. Time-dependent up-regulation of HLA-DR in HUVECs by IFN-γ.** HUVECs were treated with IFN-γ for up to 4 days and subjected to flow cytometry to assess HLA-DR expression.


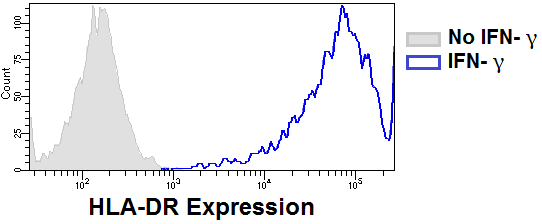


**Figure S2. HLA-DR expression levels in EA.hy926 cells.** Cell culture of the EA.hy926 cell line was treated for 4 days with IFN-γ (N = 5). The expression of HLA-DR was assessed by flow cytometry.


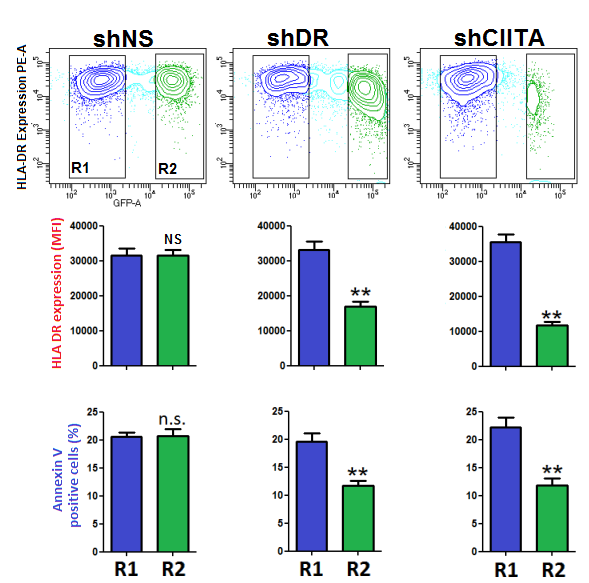


**Figure S3. HLA-DR knockdown reduced the L243-mediated ECs death.** HUVECs were transduced with GFP–containing lentiviral vectors encoding for sequencing targeting shDR or shCIITA or for a short hairpin RNA shNS (control), as described in *Materials and Methods*. After treatment with IFN-γ for 3 days cells were incubated for 3 h with L243 (N = 3). Cells were gated into R1 and R2 according to GFP-MFI levels by flow cytometry. HLA-DR antibody binding and cytotoxicity was determined in these two cell populations. Data are from a representative dot plot (upper panel) and statistical results mean ± SEM (lower panels). **P < 0.01. *GFP*, green fluorescent protein


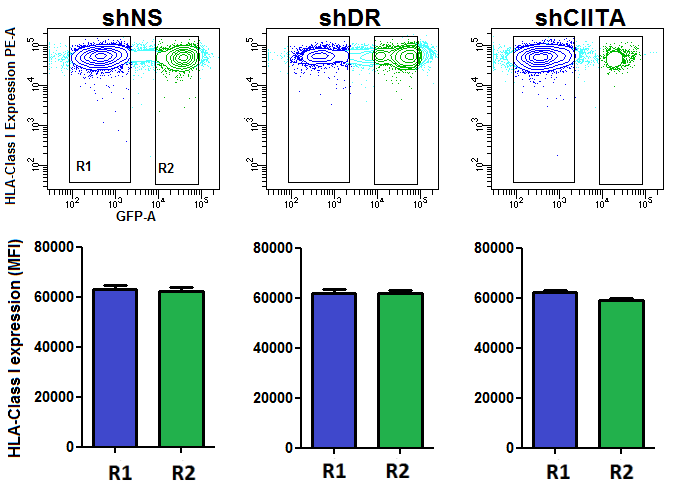


**Figure S4. Targeting HLA-DR expression by shNS, shDR or shCIITA did not interfere with HLA I expression**. HUVECs were transduced with lentiviral vectors encoding for GFP and the indicated short sequencing shDR or shCIITA or a non-specific short hairpin RNA shNS (control), as described in Materials and Methods. After treatment with IFN-γ cells were incubated for 3 h with HLA I mAb W6/32 (N = 3). Cells were gated R1 and R2 according to GFP-MFI levels by flow cytometry. HLA-DR antibody binding was determined in these two cell populations. Data are from a representative dot plot (upper panel) and statistical results mean ± SEM (lower panels).


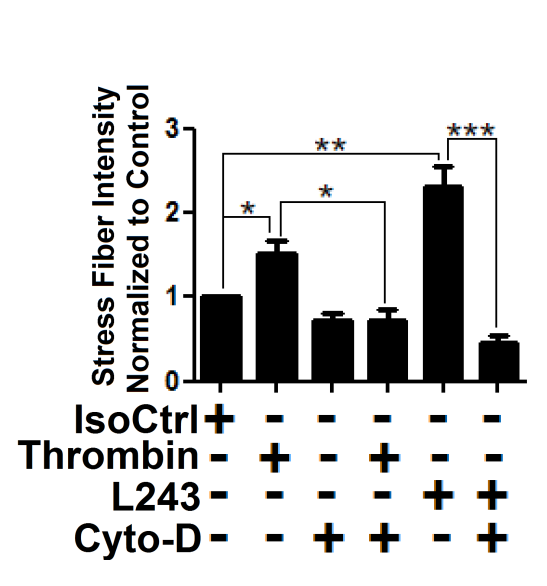


**Figure S5. Quantification of F actin stress fiber intensity.** After pretreatment with IFN-γ for 4 days, HUVECs were treated with isotype control antibody (IsoCtrl), mAb L243 or thrombin in the absence or presence of Cyto-D (100 nM) for 3 h, as indicated. Cells were then incubated with Texas Red Phalloidin for staining of F-actin stress fiber formation and DAPI for staining of nuclei (N = 3). Intensity of Phalloidin was measured in ImageJ program as described in MATERIALS AND METHODS. Data shown are as bar graphs from 3 independent experiments mean ± SEM. *P < 0.05, **P < 0.01, ***P < 0.001.


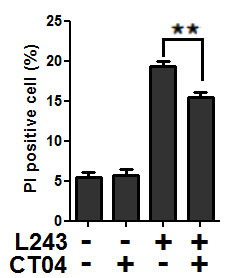


**Figure S6. Effect of Rho GTPase inhibitor CT04 on L243-mediated cytotoxicity.** HUVECs were treated with IFN-γ for 4 days, which was followed by 3 h treatment with Rho GTPase inhibitor I CT04 (1 μg/ml) and L243 (N = 3). Cytotoxicity was assessed with PI in a flow cytometer. Data are presented as mean ± SEM. **P < 0.01

**Supplementary Table**

|  | **Luminex MFI** | |
| --- | --- | --- |
| **Serum** | **HLA-DR51** | **HLA-DR52** |
| **Neg # 1** | 178 | 384 |
| **Neg # 2** | 256 | 152 |
| **HLA-DR52 # 1** | 205 | 17787 |
| **HLA-DR52 # 2** | 149 | 17534 |

**Table S1. HLA-DR antibody identification in allosera by Luminex assay.** Human allosera were analyzed by single antigen Luminex assay (One Lambda, Canoga Park, CA, USA).
